# Biocompatible and Na^+^-sensitive thin-film transistor for biological fluid sensing

**DOI:** 10.1080/14686996.2019.1656516

**Published:** 2019-08-20

**Authors:** Kensuke Ito, Hiroto Satake, Yuto Mori, Alex C. Tseng, Toshiya Sakata

**Affiliations:** Department of Materials Engineering, School of Engineering, The University of Tokyo, Tokyo, Japan

**Keywords:** Thin-film transistor, ion-sensitive membrane, biocompatibility, cytotoxicity, biological fluid sensing, 30 Bio-inspired and biomedical materials, 208 Sensors and actuators, 212 Surface and interfaces

## Abstract

In this study, we develop a Na^+^-sensitive thin-film transistor (TFT) for a biocompatible ion sensor and investigate its cytotoxicity. A transparent amorphous oxide semiconductor composed of amorphous In–Ga–Zn–oxide (a-InGaZnO) is utilized as a channel of the Na^+^-sensitive TFT, which includes an indium tin oxide (ITO) film as the source and drain electrodes and a Ta_2_O_5_ thin-film gate, onto which a Na^+^-sensitive membrane is coated. As one of the Na^+^-sensitive membranes, the polyvinyl chloride (PVC) membrane with bis(12-crown-4) as the ionophore used on the TFT sensors shows good sensitivity and selectivity to changes in Na^+^ concentration but has high cytotoxicity owing to the leaching of its plasticizer to the solution; the plasticizer is added to solve and entrap the ionophore in the PVC membrane. On the other hand, a plasticizer-free Na^+^-sensitive membrane, the fluoropolysilicone (FPS) membrane with the bis(12-crown-4) ionophore, also reduces cell viability owing to the leaching of the ionophore. However, the FPS membrane with calix[4]arene as the ionophore on the gate of TFT sensors exhibits not only favorable electrical properties but also the lack of cytotoxicity. Thus, considering structural flexibility of TFTs, a platform based on TFT sensors coated with the Na^+^-sensitive FPS membrane containing calix[4]arene is suitable as a biocompatible Na^+^ sensing system for the continuous monitoring of ionic components in biological fluids such as sweat and tears.

## GRAPHICAL ABSTRACT

**Figure:**
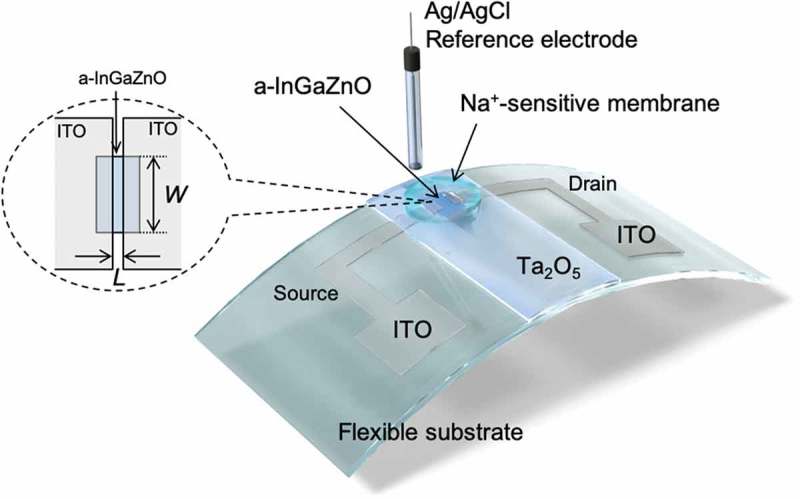

## 1. Introduction

Ion-sensitive membranes (ISMs) have been widely developed worldwide to selectively transduce ionic charges on detection devices. Most of them were proposed for environmental and biomedical applications [1–11]. In particular, Na^+^-sensitive membranes were utilized to examine the intrinsic excitability of nerve cells and the change in Na^+^ concentration in sweat on potentiometric detection devices such as ion-sensitive field-effect transistors (ISFETs) [12,13]. However, polyvinyl chloride (PVC)-based ISMs often include a plasticizer to disperse the ionophore in the PVC membrane. There is concern that the leaching of the plasticizer into a solution may cause cytotoxicity when an ISM-coated sensor is used for a long time (>1 day) to monitor health condition in daily life. This concern was not addressed in a previous work because of short-term measurements (<2 h at most)[13]. On the other hand, a previous paper mentioned that the lifetime of ISFETs was limited by the leaching of the plasticizer out of the PVC membrane, which resulted in the poor adhesion of the membrane to the gate surface [3]. Considering the mobility and dispersibility of the ionophore in the membrane without a plasticizer, a matrix material that has a glass transition temperature below room temperature and has polarity should be selected. In addition, the dielectric constant of the polymer used is desired to be in the range of 4–15 so as not to increase membrane resistance, for example, fluoropolysilicone (FPS) [2,10,11]. Thus, an FPS-based ion-sensitive membrane without a plasticizer may be utilized as a matrix for biocompatible Na^+^-sensitive sensors.

Most biological phenomena are closely related to ionic behaviors such as ion channel activities at the cell membrane or behaviors of various ions in biological fluids. Therefore, the direct detection of ionic charges enables the straightforward analysis of various biological events, which contributes to the development of a simple and quick test for the early finding and prevention of diseases. The operation of FET-based biosensors is based on the potentiometric measurement of biomolecular recognition events, which are specifically detected by modifying the gate sensing surface with various functional membranes such as ion-sensitive oxides and polymers [14,15]. That is, the changes in ionic or molecular charges caused by biological phenomena at/in such membranes contribute to the changes in the electrical properties of FET-based biosensors; therefore, FET biosensors have a significant advantage in that they enable the direct monitoring of biomolecular recognition events on the gate without using materials for labeling DNA [16–19], proteins [20,21], cells [22–24], and small biomarkers [25–27]. Moreover, a thin-film transistor (TFT) device contributes to the flexibility and stretchability [28,29] of the biosensors for the *in situ* monitoring of biological fluids such as sweat on the skin. Similarly to the FET biosensors, TFT devices have been applied to biosensors recently [30–32]. Although some new semiconducting materials such as graphene and MoS_2_ have been recently utilized as the channel of FETs for biosensing [33–35], amorphous In–Ga–Zn oxide (a-InGaZnO) has been used as the channel of H^+^-sensitive TFTs [as an example of ion-sensitive TFTs (ISTFTs)] for long-term, real-time, label-free, and noninvasive monitoring of cellular functions, which requires noncytotoxic materials [32]. For a-InGaZnO-channel ISTFT sensors, in particular, a SiO_2_ thin film, which was coated by sputtering, was utilized as the gate insulator and showed a pH responsivity (about 56 mV/pH) near the ideal Nernstian response at 25°C. This is why cellular respiration is monitored as the change in pH at the cell/gate interface using ISTFT sensors [24]. The oxide membranes used as a gate insulator, such as SiO_2_ and Ta_2_O_5_, are the basic configuration of ISTFT sensors similarly to ISFET sensors [36]. Thus, a-InGaZnO-channel ISTFT sensors can be used as Na^+^-sensitive and flexible biosensors, but such sensors have not yet been developed. Moreover, there has been no research on the cytotoxicity of plasticizer-free FPS membranes in relation to their applications in flexible and biocompatible Na^+^-sensing systems for the continuous monitoring of ionic components in biological fluids such as sweat and tears.

In this study, we develop an a-InGaZnO-channel ISTFT sensor integrated with Na^+^-sensitive membranes for use in a biocompatible and flexible Na^+^-sensitive TFT sensor. In particular, the electrical properties and cytotoxicity of Na^+^-sensitive membranes are investigated in the presence or absence of a plasticizer in the membranes. As the Na^+^-sensitive membranes, PVC, which requires a plasticizer to dissolve ionophores, and a plasticizer-free FPS are used as the base materials, which are mixed with ionophores such as crown ether and calixarene.

## 2. Experimental section

### 2.1. Chemicals

The following chemicals were used in this study. 2-Nitrophenyl octyl ether (NPOE), bis[(12-crown-4)methyl] 2-dodecyl-2-methylmalonate [bis(12-crown-4)], tetrakis[3,5-bis(trifluoromethyl)phenyl]borate sodium salt (Na-TFPB), 4-(2-hydroxyethyl)-1-piperazineethanesulfonic acid (HEPES), and tetrahydrofuran (THF) were purchased from Dojindo Molecular Technologies Inc. Tetrakis-(4-chlorophenyl)-borate potassium salt (K-TCPB) was purchased from Alfa Aesar. 4-tert-butylcalix[4]arene-tetraacetic acid tetraethyl ester (calix[4]arene) was purchased from Sigma Aldrich. PVC, sodium dihydrogen phosphate (NaH_2_PO_4_), sodium hydrogen phosphate (Na_2_HPO_4_), citric acid, potassium chloride (KCl), phosphate-buffered saline (PBS), and pH standard buffer solutions were purchased from Wako Pure Chemicals Industries, Ltd. Fluorosilicone gel (FE-73) was purchased from Shin-Etsu Chemical Co., Ltd.

### 2.2. Structural concept of ISTFT

**Figure 1.**
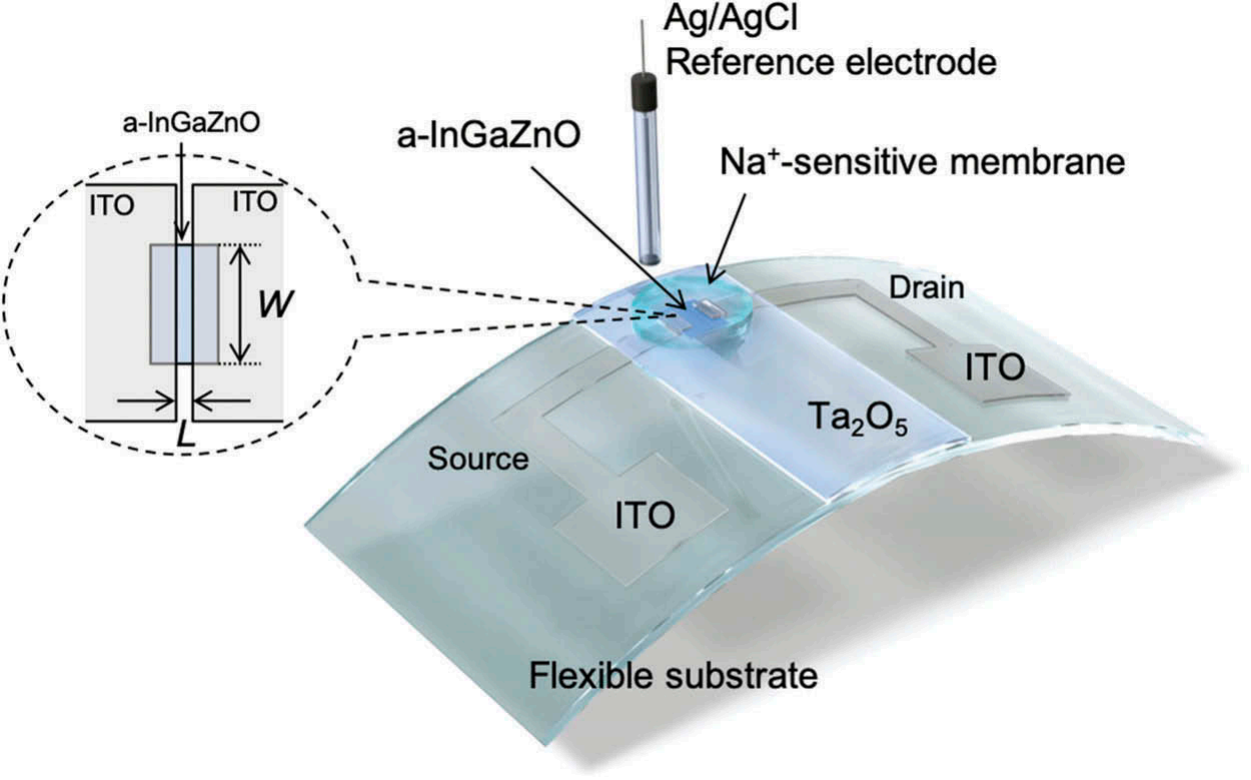
Structure of ISTFT. The schematic illustration also shows the setup for measurement.

All the ISTFT thin films used in this study were fabricated on a glass substrate or a polyimide (PI) flexible film (DuPont^TM^ Kapton®) by sputtering (ULVAC, Inc.). A TFT based on a-InGaZnO, which was sputtered with 5% O_2_, was utilized to detect ion charges based on biological phenomena. Indium tin oxide (ITO) was used as conductive electrodes such as the source and drain, and tantalum oxide (Ta_2_O_5_) was thinly coated as the gate insulator, functioning as the H^+^-sensitive membrane, as shown in Figure 1. This is why a Ta_2_O_5_ membrane has pH-responsive hydroxyl groups at the surface in a solution [36]. The sputtered films were heat treated at 450°C. Fundamentally, the carrier mobility of the fabricated device was approximately 14 cm_2_/Vs, which was determined from the electrical properties of ISTFT devices (Figure S1) and was similar to those obtained in previous works [37,38]. The thicknesses of InGaZnO, ITO, and Ta_2_O_5_ were 100, 100, and 100 nm, respectively. The width (*W*) and length (*L*) of the gate channel, which was fabricated using a lithography technology, were 360 and 12 μm, respectively. A transparent polycarbonate ring (inner diameter 10 mm, outer diameter 12 mm) was encapsulated on the device using an epoxy resin (ZC-203T, Pelnox Ltd., Japan) except for the sensing surface. AgCl-coated Ag was utilized as the reference electrode in an oversaturated KCl solution, which was electrically connected to a sample solution through a porous glass or an agar-based salt bridge.

### 2.3. Preparation of Na^+^-sensitive membrane

200 mg of NPOE as a plasticizer, 10 mg of bis(12-crown-4) as an ionophore, 1.1 mg of K-TCPB as an agent for anion exclusion, and 101 mg of PVC as a base material were mixed in 3 mL of THF, and 262 μL of the mixed solvent was dropped onto the gate surface in the polycarbonate ring (Figure 1). Subsequently, THF was vaporized in air ambient overnight and then the Na^+^-sensitive PVC membrane was conditioned in a 0.1 M NaCl solution overnight. On the other hand, a plasticizer-free Na^+^-sensitive membrane was prepared as follows. After mixing 3.0 mg of bis(12-crown-4) or 4.5 mg of calix[4]arene as an ionophore, 2.0 mg of Na-TFPB as an agent for anion exclusion, and 150 mg of FE-73 as a base material in 750 μL of THF, 10 μL of the mixed solvent was dropped onto the gate surface and then heated at 120°C for 2 h in air ambient (Figure 1). The heat treatment was performed to induce the addition reaction of hydrosilylation for polymerization.

### 2.4. Measurement of electrical signals with Na^+^-sensitive TFT

The detection principle of solution-gate ISTFT is based on the potentiometric detection of changes in charge density at the gate electrode, on which specific bindings are induced for molecular recognitions. The ionic charges at the gate insulator, the Ta_2_O_5_ film, interact electrostatically with electrons at the a-InGaZnO channel across the thin gate insulator and contribute to electrical signals through the field effect, resulting in a change in the source–drain current (*I*_D_) at the channel. The electrical properties of TFT devices, such as a gate voltage (*V*_G_)–*I*_D_ electrical characteristic and a change in the surface potential at the gate, were analyzed in buffer solutions to investigate the potential responses to the change in the concentration of cations, such as H^+^, Na^+^, and K^+^ (pH, pNa, and pK), using a semiconductor parameter analyzer (B1500A, Agilent Technologies) and a real-time potentiometric analyzer (Optgenesys Co., Ltd.), respectively. To investigate pH responsivity, phosphate buffer solutions were prepared with pHs from 5.81 to 8.11 by controlling the mixing ratio of Na_2_HPO_4_ to NaH_2_PO_4_. Also, the standard buffer solutions with pHs of 4.01, 6.86, 7.41, 9.18, and 10.01 were prepared. Moreover, the concentration of Na^+^ (or K^+^) was varied from 1 mM to 1 M by adding an adequate volume of 1 M NaCl (or 1 M KCl) to 10 mL of 50 mM HEPES + 14.875 mL of deionized water + 125 μL of 1 M NaOH (or 1 M KOH) at a constant pH of 7.2. To evaluate basic electrical signals, the shift in the threshold voltage Δ*V*_T_ was defined as the difference in *V*_G_ at a constant *I*_D_ in the *V*_G_–*I*_D_ characteristics. The *V*_G_–*I*_D_ characteristics were measured ten times to determine the change in the concentration of cations in this study. The surface potential at the gate surface was monitored using a circuit [23] with which the change in gate surface potential can be read out directly at a constant *I*_D_ (Figure S2). In this study, the drain voltage (*V*_D_) and *I*_D_ were set to be 0.25 V and 25 μA, respectively.

### 2.5. Evaluation of cytotoxicity

SKOV-3 (an ovarian cancer cell line) cells were cultured in Dulbecco’s modified Eagle’s medium (DMEM, Gibco) with 1 g/L glucose and 10% fetal bovine serum (FBS) including 50 U/mL penicillin and 50 μg/mL streptomycin on a conventional cell culture dish (Falcon® cell culture dishes) in an incubator (37°C, 5% CO_2_) for 3 days. The number of cells seeded on the membranes was controlled to 2.5 × 10_5_ cells/mL. After trypsin (Gibco) treatment to peel off the cells from the dish, 1.0 × 10_4_ cells/cm_2_ were then transferred onto the Na^+^-sensitive membranes composed of PVC or FPS in the polycarbonate ring, where the culture medium was added and maintained at 37°C and 5% CO_2_ for 1 and 3 days for the evaluation of cytotoxicity.

The number of viable cells on the membrane was quantified by absorbance measurement (435 nm) on the basis of the cleavage of water-soluble tetrazolium salts (WST-1) by mitochondrial dehydrogenase in the viable cells. For the WST-1 test, 16.3 mg of WST-1 was added to 4.5 mL of 20 mM HEPES buffer (pH 7.4) containing 500 μL of 2 mM 1-methoxy-PMS. Then, the volume ratio of the WST solution to the cultured medium was controlled to 1:10 and the mixture solution was maintained at 37°C and 5% CO_2_ for 3 h. Moreover, the amount of lactate dehydrogenase (LDH) released from cells with a damaged cytoplasmic membrane was measured to examine the number of dead or damaged cells. In this case, WST-1 was reduced by NaDH^+^/H^+^, which was generated by the reaction between LDH and oxidized beta-nicotinamide adenine dinucleotide (β-NAD^+^), resulting in the formation of formazan dye. To measure the amount of LDH released, the mixture of 12 mg/mL sodium lactate, 1 mg/mL β-NAD^+^, 100 µg/ml 1-methoxy-PMS, 1 mg/mL WST-1, 1 mg/mL bovine serum albumin (BSA), and 4 mg/mL sucrose was prepared in PBS, 50 μL of which was mixed with 50 μL of the culture medium. Then, the amount of LDH released was quantified by absorbance measurement (435 nm) of the formazan dye.

## 3. Results and discussion

**Figure 2.**
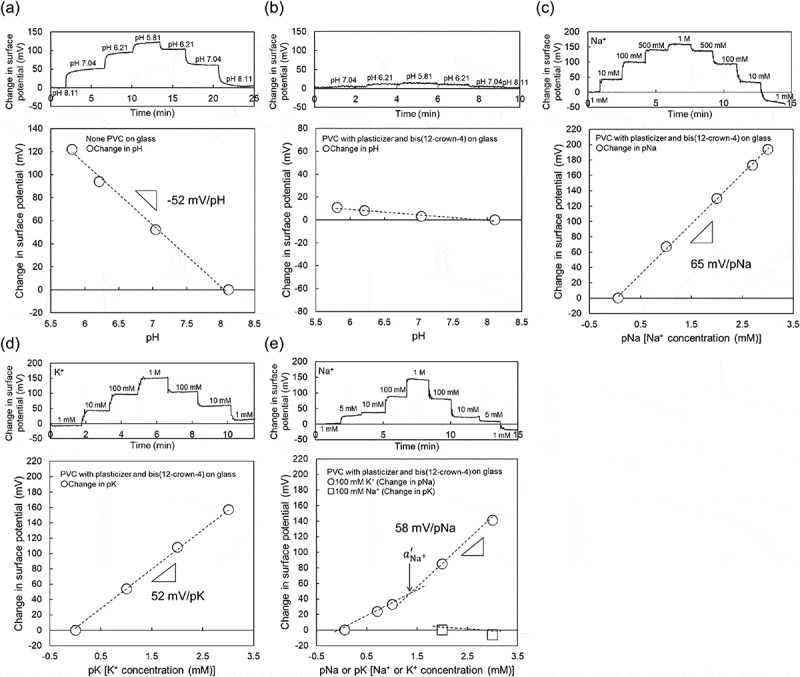
Changes in surface potential (Δ*V*_out_) at the gate and calibration curve of Na^+^-sensitive PVC-gate TFT on a glass substrate. (a) pH response of Ta_2_O_5_-gate ISTFT. (b) pH response of Na^+^-sensitive PVC-gate TFT. (c) pNa response of Na^+^-sensitive PVC-gate TFT. (d) pK response of Na^+^-sensitive PVC-gate TFT. (e) pNa response of Na^+^-sensitive PVC-gate TFT in a buffer with 100 mM K^+^ and pK response of the device in a buffer with 100 mM Na^+^. In (a) and (b), the surface potentials at pH 8.11 were offset to be zero. In (c), the surface potential at 1 mM Na^+^ was offset to be zero. In (d), the surface potential at 1 mM K^+^ was offset to be zero. In (e), the surface potentials at 1 mM Na^+^ and 100 mM K^+^ were offset to be zero.

**Figure 3.**
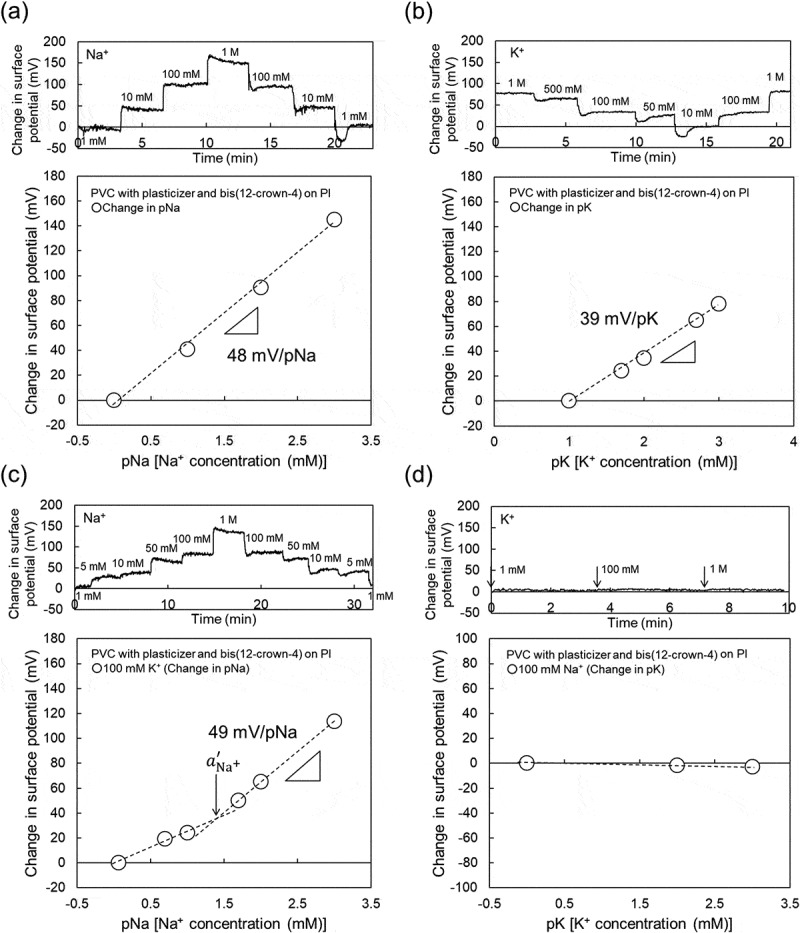
Changes in surface potential (Δ*V*_out_) at the gate and calibration curve of Na^+^-sensitive PVC-gate TFT on a PI flexible film. (a) pNa response of Na^+^-sensitive PVC-gate TFT. (b) pK response of Na^+^-sensitive PVC-gate TFT. (c) pNa response of Na^+^-sensitive PVC-gate TFT in a buffer with 100 mM K^+^. (d) pK response of the device in a buffer with 100 mM Na^+^. In (a) and (c), the surface potentials at 1 mM Na^+^ were offset to be zero. In (b), the surface potential at 10 mM K^+^ was offset to be zero. In (d), the surface potential at 1 mM K^+^ was offset to be zero.

**Figure 4.**
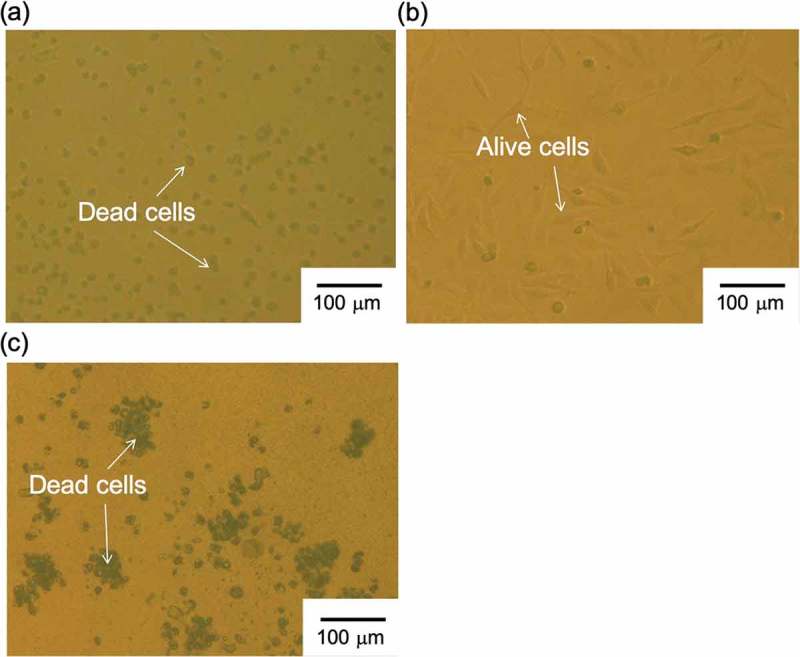
Photographs of SKOV-3 cells cultured on PVC membrane (a) with and (b) without bis(12-crown-4), NPOE, and K-TCPB and (c) with NPOE only. SKOV-3 cells were cultured on each substrate for 54 h.

As shown in a previous work, the oxide-gate ISTFT sensor showed an ideal pH responsivity close to the Nernstian response (59.1 mV/pH at 25°C) [32]. This is because the oxide-gate surface of the ISTFT was covered by hydroxyl groups (silanol groups) in a solution, which were sensitive to H^+^, resulting in the pH response [36]. Figure 2 shows the electrical properties of ISTFT on a glass substrate. The Ta_2_O_5_-gate ISTFT used in this study showed a pH sensitivity of about 53 mV/pH, which is similar to that obtained in a previous work, as shown in Figure 2(a). However, such a pH response of ISTFT was hardly found when the Na^+^-sensitive PVC membrane was coated on the oxide-gate surface, as shown in Figure 2(b). Importantly, the Na^+^-sensitive PVC-gate TFT not only showed high sensitivity to changes in Na^+^ concentration in the range from 1 mM to 1 M (Figure 2(c)), which was about 64 mV/pNa close to the Nernstian response, but also contributed to the K^+^ response of about 53 mV/pK at K^+^ concentrations from 1 mM to 1 M (Figure 2(d)). The selectivity coefficient of bis(12-crown-4) included in the PVC membrane to Na^+^ in a solution containing K^+^ was reported to be about 1/100 [39]. This is why the Na^+^ sensitivity of the PVC-gate TFT was investigated at various Na^+^ concentrations from 1 mM to 1 M in the buffer solution containing 100 mM K^+^ at a constant pH of 5.5. As shown in Figure 2(e), the Na^+^ sensitivity was almost maintained at various Na^+^ concentrations even in the buffer solution with 100 mM K^+^, although the sensitivity seemed to be slightly low at Na^+^ concentrations below 10 mM. Considering the calibration curve shown in Figure 2(c), two different calibration curves were fitted in Figure 2(e) to analyze the effect of K^+^ as an interfering ion on Na^+^. To calculate the selectivity coefficient $K_{Na^{+},K^{+}}$, the Nernstian potential ψ is modified using the following Nicolsky–Eisenman equation:

(1) $$\psi =const.+\frac{2.303RT}{n_{Na^{+}}F}log\left(a_{Na^{+}}+\sum_{j}K_{Na^{+},K^{+}}a_{K^{+}}\frac{n_{Na^{+}}}{n_{K^{+}}}\right),$$

where $a_{Na^{+}}$ and $a_{K^{+}}$ are the activities of Na^+^ and K^+^, and $n_{Na^{+}}$ and $n_{K^{+}}$ are the valences of Na^+^ and K^+^, respectively. Here, $K_{Na^{+},K^{+}}$ is expressed on the basis of the fixed interference method (FIM) as described in a previous paper [40] as Equation 2,

(2) $$K_{Na^{+},K^{+}}=\frac{{a_{Na^{+}}}^{\prime }}{a_{K^{+}}^{const}}$$

where $a_{K^{+}}^{const}$ is the constant activity of the interfering ion K^+^, which was 100 mM in this study. The value of ${a_{Na^{+}}}^{\prime }$ is calculated from the intersection of the extrapolation of the linear portions in Figure 2(e). As a result, $K_{Na^{+},K^{+}}$ was calculated to be about 1.8 × 10^−2^, which indicated the expected selectivity of bis(12-crown-4) to Na^+^. Moreover, the Na^+^ sensitivity of the PVC-gate TFT was investigated at various K^+^ concentrations from 100 mM to 1 M (relatively high concentration) in the buffer solution containing 100 mM Na^+^ at a constant pH of 5.5 (Figure 2(e)). From Figure 2(e), no pK sensitivity was found owing to the presence of Na^+^ in the solution. Thus, the Na^+^-sensitive PVC-gate TFT on the glass showed good electrical properties such as sensitivity and selectivity to the changes in pNa. Moreover, the ISTFT sensor was coated on a PI flexible film. In this case, the Na^+^-sensitive PVC-gate TFT on the PI flexible film also showed a relatively high sensitivity to the changes in Na^+^ concentration in the range from 1 mM to 1 M (Figure 3(a)), which was about 48 mV/pNa, while contributing to the K^+^ response of about 39 mV/pK at K^+^ concentrations from 10 mM to 1 M (Figure 3(b)). In particular, $K_{Na^{+},K^{+}}$ was calculated to be about 3.3 × 10^−2^ from Figure 3(c), which indicated the sufficient selectivity of bis(12-crown-4) to Na^+^ even when using the Na^+^-sensitive PVC-gate TFT coated on the PI flexible film. Also, the Na^+^-sensitive PVC-gate TFT did not show the K^+^ response at K^+^ concentrations from 100 mM to 1 M (relatively high concentrations) in the buffer solution containing 100 mM Na^+^ at a constant pH of 5.5 (Figure 3(d)). Thus, the Na^+^-sensitive PVC-gate TFT showed high sensitivity and selectivity to Na^+^ even on the flexible film. However, its biocompatibility was low. Figure 4(a,b) show SKOV-3 cells cultured on the Na^+^-sensitive PVC membrane or the original PVC membrane without other chemicals coated on the cell culture dish. SKOV-3 cells cultured for 54 h in the former dish were clearly observed to be dead (Figure 4(a)), while those in the latter dish were alive (Figure 4(b)). Moreover, the cell culture medium, in which the Na^+^-sensitive PVC membrane was immersed for 54 h, caused cell death even when the SKOV-3 cells were cultured directly on the dish. In particular, SKOV-3 cells were dead on the PVC membrane with NPOE only. This means that the cytotoxicity of the plasticizer was found for SKOV-3 cells, as expected (Figure 4(c)). That is, the organic solvents such as the plasticizer and ionophores leaked into the culture medium, resulting in the cytotoxicity of the medium. Therefore, a plasticizer-free Na^+^-sensitive membrane from which the ionophore does not leak should be developed for use in biocompatible ion sensors.

**Figure 5.**
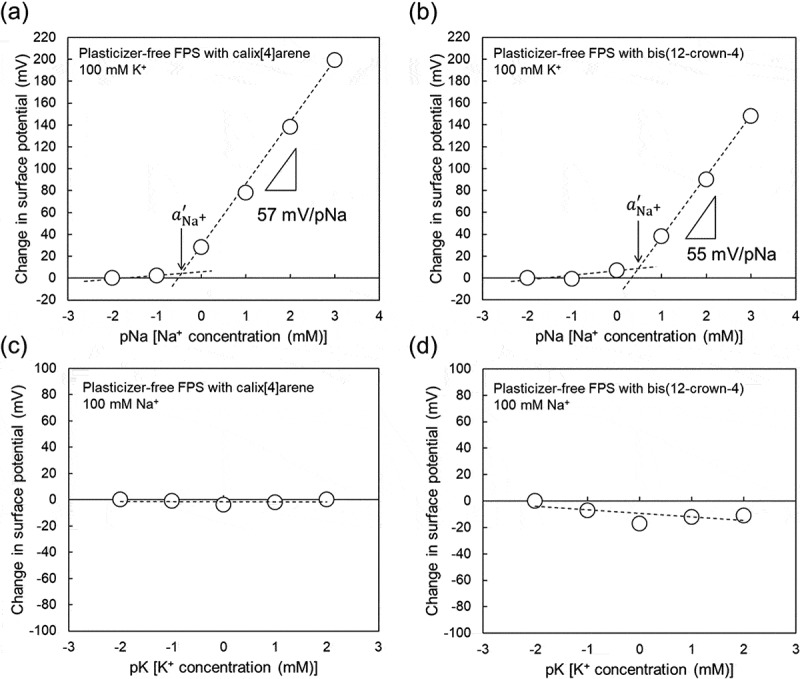
Calibration curve based on change in surface potential (Δ*V*_out_) at the gate with plasticizer-free Na^+^-sensitive FPS-gate TFT on a glass substrate. (a) pNa response of plasticizer-free Na^+^-sensitive FPS-gate TFT in a buffer with 100 mM K^+^. Calix[4]arene was used as the ionophore. Reproducible data of pNa sensitivity from 55 to 59 mV/pNa and the selectivity coefficient $K_{Na^{+},K^{+}}$ from 4.8 × 10^−3^ to 4.4 × 10^−2^ as shown in (a) were obtained (n = 3). (b) pNa response of plasticizer-free Na^+^-sensitive FPS-gate TFT in a buffer with 100 mM K^+^. Bis(12-crown-4) was used as the ionophore. Reproducible data of pNa sensitivity from 53 to 57 mV/pNa and the selectivity coefficient $K_{Na^{+},K^{+}}$ from 2.7 × 10^−2^ to 5.8 × 10^−2^ as shown in (b) were obtained (n = 3). (c) pK response of plasticizer-free Na^+^-sensitive FPS-gate TFT in a buffer with 100 mM Na^+^. Calix[4]arene was used as the ionophore. (d) pK response of plasticizer-free Na^+^-sensitive FPS-gate TFT in a buffer with 100 mM Na^+^. Bis(12-crown-4) was used as the ionophore. In (a) and (b), the surface potentials at 10 μM Na^+^ were offset to be zero. In (c) and (d), the surface potentials at 10 μM K^+^ were offset to be zero.

Here, a plasticizer-free FPS membrane was applied in a Na^+^-sensitive TFT sensor. FE-73 was hydrosilylated for its polymerization as a base material at 120°C for 2 h in air ambient, including the agent for anion exclusion and bis(12-crown-4) or calix[4]arene. FE-73 has a glass transition temperature below room temperature (gel remains consistent from −60 to 150°C) and a dielectric constant of 7.0 (50 Hz) and shows polarity owing to fluoro groups. Thus, the plasticizer-free FPS membrane can be obtained by the polymerization of FE-73, enabling the dispersion of ionophores in the membrane without a plasticizer, for application in the Na^+^-sensitive TFT sensor. Figure 5 shows the electrical properties of the plasticizer-free Na^+^-sensitive FPS-gate TFT on a glass substrate. Similarly to the Na^+^-sensitive PVC-gate TFT, the plasticizer-free Na^+^-sensitive FPS-gate TFT with bis(12-crown-4) or calix[4]arene responded to changes in not only Na^+^ concentration but also K^+^ concentrations. Moreover, it showed a high sensitivity of approximately 60 mV/pNa at Na^+^ concentrations of more than 1 mM even when 100 mM K^+^ was included in the buffer (Figure 5(a,b)), but it hardly responded to changes in K^+^ concentration when only 100 mM Na^+^ was included in the buffer (Figure 5(c,d)), regardless of the type of ionophore included in the membrane. The selectivity coefficients, $K_{Na^{+},K^{+}}$, were calculated using Equations 1 and 2 to be about 4.8 × 10^−3^ for the calix[4]arene-containing membrane and about 2.7 × 10^−2^ for the bis(12-crown-4)-containing membrane. These results of the plasticizer-free Na^+^-sensitive FPS-gate TFT with the ionophores on the glass are also expected on the PI film substrate similarly to the Na^+^-sensitive PVC-gate TFT. Therefore, the plasticizer-free FPS based on FE-73 can be applied as the Na^+^-sensitive and selective membrane on the gate surface of TFT devices. When such devices are used to detect pNa in a whole sample, a basic output signal, which is defined in a pNa standard solution with pNa more than ${a_{Na^{+}}}^{\prime }$ before the measurement, should be set as the initial point when the electrical measurement is started.

**Figure 6.**
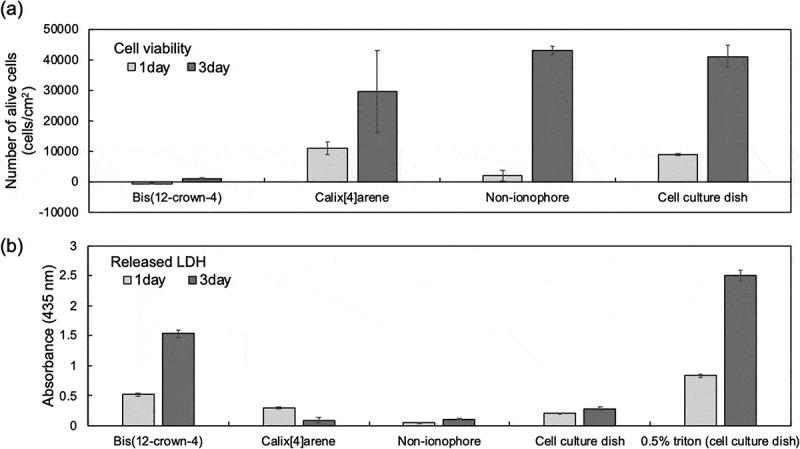
Cytotoxicity test. (a) Number of viable cells determined by the WST test. Four substrates for cell culture were prepared: plasticizer-free FPS membrane with bis(12-crown-4), plasticizer-free FPS membrane with calix[4]arene, plasticizer-free FPS membrane without ionophores, and cell culture dish without membranes. (b) Evaluation of LDH released from cells with damaged cytoplasmic membrane. Five culture conditions were set for cell culture: plasticizer-free FPS membrane with bis(12-crown-4), plasticizer-free FPS membrane with calix[4]arene, plasticizer-free FPS membrane without ionophores (control), cell culture dish without membranes (control), and cell culture dish without membranes to which 0.5% Triton X-100, which induces damage to the cell membrane, was added (control).

Lastly, the biocompatibility of the plasticizer-free FPS membrane with or without ionophores was evaluated by the cytotoxicity test. The number of living cells and the amount of LDH released by the damaged cytoplasmic membrane were investigated to evaluate the cytotoxicity of the plasticizer-free FPS membrane, as shown in Figure 6. On the basis of these two cytotoxicity parameters, almost all of the SKOV-3 cells cultured on the plasticizer-free FPS membrane with bis(12-crown-4) was found to be dead after culturing for only 1 day, whereas those cultured on the plasticizer-free FPS membrane with calix[4]arene were alive and the number of cells increased after culturing for 3 days, compared with the control culture. That is, calix[4]arene remained in the FPS membrane for 3 days, whereas bis(12-crown-4) leached out from the FPS membrane into the culture medium in 1 day. Also, the pNa sensitivity and $K_{Na^{+},K^{+}}$ of the plasticizer-free Na^+^-sensitive FPS-gate TFT with calix[4]arene were sufficiently maintained at approximately 60 mV/pNa and less than about 10^−2^ order, respectively, for 10 days, as shown in Figure S3. This means that calix[4]arene molecules are not released in solutions even when the device is repeatedly used for 10 days. In addition, sufficient pNa sensitivity and selectivity were obtained at Na^+^ concentrations with pNa more than ${a_{Na^{+}}}^{\prime }$ (about 1 mM) even when the concentration of K^+^ for interference was as high as 100 mM (Figure 5(a)). In human insensible perspiration, the K^+^ concentration is in the range of 1–10 mM, whereas the Na^+^ concentration is about 50 mM [41–43]. That is, the change in the Na^+^ concentration in secreted sweat can be selectively detected using the plasticizer-free Na^+^-sensitive FPS-gate TFT with calix[4]arene. Therefore, the plasticizer-free Na^+^-sensitive FPS-gate TFT with calix[4]arene is suitable for a biocompatible ion-sensing system, considering Figures 5 and 6. In addition, the structural advantage of TFT devices contributes to their flexibility for the monitoring of Na^+^ in sweat *in situ*. On the other hand, to prevent the leaching of ionophores such as bis(12-crown-4), the density of cross linking and other factors should be controlled to entrap ionophores in polymerized membranes. Alternatively, ionophores with reactive groups may be copolymerized with a base material.

## 4. Conclusions

In this study, we developed a biocompatible and flexible Na^+^-sensitive TFT sensor, the channel of which was composed of a-InGaZnO, and evaluated its cytotoxicity. The Na^+^-sensitive membrane-coated gate TFT sensors showed high sensitivity (ca. 60 mV/pNa) and selectivity ($K_{Na^{+},K^{+}}$ < ca. 10^−2^), which were the same as those obtained by the silicon-based ISFET sensors [10]. However, the PVC-based Na^+^-sensitive membrane with a plasticizer had high cytotoxicity to cultured cells owing to the leaching of the plasticizer. To overcome this problem, the plasticizer-free FPS-based Na^+^-sensitive membrane was coated on the gate of the TFT sensor. As a result, the modified TFT sensor showed biocompatibility and high sensitivity and selectivity to Na^+^, only when calix[4]arene as an ionophore was used in the membrane. Moreover, the pNa sensitivity and $K_{Na^{+},K^{+}}$ of the plasticizer-free Na^+^-sensitive FPS-gate TFT with calix[4]arene were sufficiently maintained around 60 mV/pNa and less than about 10^−2^ order, respectively, for 10 days, although further investigations on how calix[4]arene molecules disperse in the plasticizer-free FPS membrane may be necessary. The remaining problem is that other ionophores such as bis(12-crown-4) can leach out from even the FPS membrane. This is why the density of cross linking and other factors should be controlled to more strictly entrap ionophores in polymerized membranes, or ionophores with reactive groups may be copolymerized with a base material, which should be further studied in the future. One of the important points for developing a biocompatible ion sensor is to arrange a base material of the ion-sensitive membrane that retains ionophores for a long term, which ensures the prevention of cytotoxicity. Indeed, plasticizers such as NPOE should not be utilized for biological sensing owing to their cytotoxicity.
