# Supplementary material for: Biocompatible and Na+-sensitive thin-film transistor for biological fluid sensing
Source: Sci Technol Adv Mater. 2019 Aug 20;20(1):917–26. doi: 10.1080/14686996.2019.1656516 (PMC6764347; doi:10.1080/14686996.2019.1656516)
Supplement: Supplemental Material [file TSTA_A_1656516_SM5604.docx]

**Supporting Information**

**Biocompatible and Na^+^-sensitive thin film transistor for biological fluid sensing**

Kensuke Ito, Hiroto Satake, Yuto Mori, Alex C. Tseng, and Toshiya Sakata^*^

Department of Materials Engineering, School of Engineering, The University of Tokyo, 7-3-1 Hongo, Bunkyo-ku, Tokyo, Japan 113-8656

*E-mail T.S.: sakata@biofet.t.u-tokyo.ac.jp

**S1. *V*_G_-*I*_D_ and *V*_D_-*I*_D_ electrical characteristics of ISTFT**

The electrical properties of TFT devices such as the *V*_G_-*I*_D_ and *V*_D_-*I*_D_ electrical characteristics were analyzed in the PBS buffer (pH 7.4) using a semiconductor parameter analyzer (B1500A, Agilent Technologies), as shown in **Figure S1**. The carrier mobility *μ*_sat_ developed in this study was calculated as approximately 14 cm^2^/Vs using the following equation,

$$\mu_{\mathrm{sat}}=\frac{2L{(\sqrt{I_{D1}}-\sqrt{I_{D2}})}^{2}}{C_{\mathrm{OX}}W{(V_{G1}-V_{G2})}^{2}}$$

where the width (*W*) and length (*L*) of the gate channel were 360 μm and 12 μm, respectively, *C*_OX_ was measured as 2.21 × 10^2^ nF (50 Hz) using a impedance analyzer (Agilent Technologies), and *V*_G1_, *V*_G2_, *I*_D1_, and *I*_D2_ were estimated as 1.25 V, 0.75 V, 40.5 μA, and 6.73 μM from **Figure S1b**, respectively.


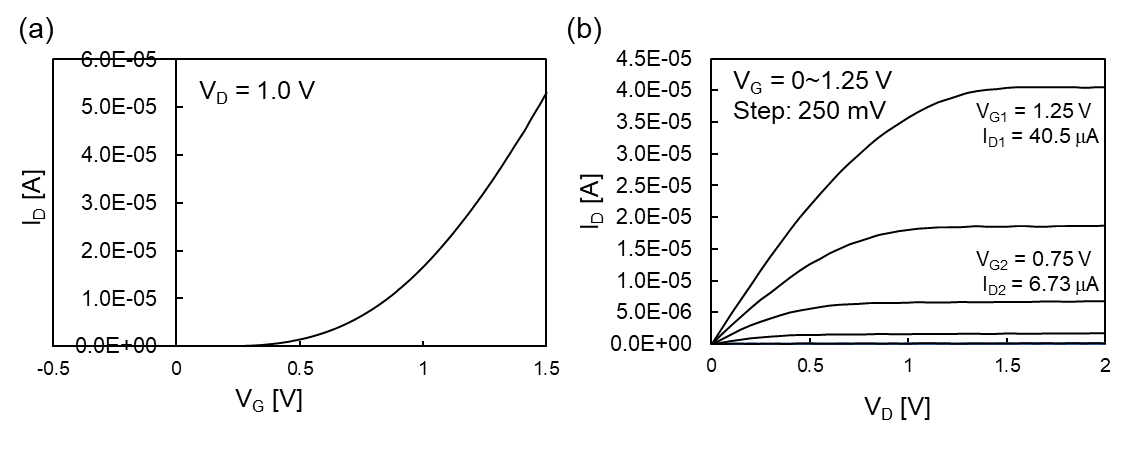


**Figure S1** *V*_G_-*I*_D_ and *V*_D_-*I*_D_ electrical characteristics of ISTFT used in this study. No membrane was coated on the Ta_2_O_5_ gate insulator.

**S2. Source follower circuit for FET real-time measurement**

The change in surface potential (Δ*V*_out_) at the gate was measured at a constant I_D_ (700 μA) using the source follower circuit shown in **Figure S2**; thus, the detected Δ*V*_out_ was regarded as the change in the source-gate voltage (Δ*V*_S_), which was equal to –Δ*V*_T_. Using this system, the surface potential of the FET can be monitored in real time.

**Figure S2** Electrical circuit (source follower circuit) for measuring surface potential of FET sensor.^S1^

**S3. Reusability of plasticizer-free Na^+^-sensitive FPS-gate TFT with calix[4]arene**

The pNa sensitivity and the selectivity coefficient $K_{\mathrm{Na}^{+},K^{+}}$ of the plasticizer-free Na^+^-sensitive FPS-gate TFT with calix[4]arene were measured for 10 days. As a result, the pNa sensitivity and $K_{\mathrm{Na}^{+},K^{+}}$ were sufficiently maintained around 60 mV/pNa and less than about 10^-2^ order, respectively, for 10 days, as shown in **Figure S3**. Therefore, we have demonstrated the sufficient reusability of the devices for 10 days.


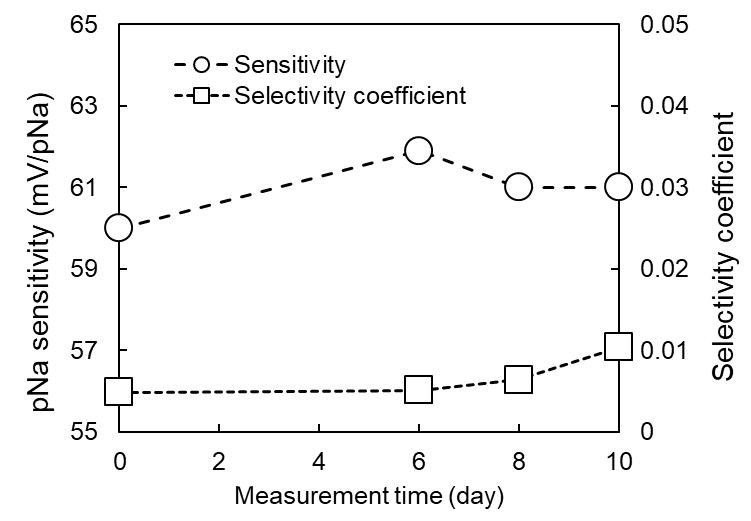


**Figure S3** pNa sensitivity and selectivity coefficients $K_{\mathrm{Na}^{+},K^{+}}$ of plasticizer-free Na^+^-sensitive FPS-gate TFT with calix[4]arene.

**References**

1. Sakata, T.; Kamahori, M.; Miyahara, Y. DNA analysis chip based on field-effect transistors. *Jpn. J. Appl. Phys.* **2005**, *44*, 2854.
